# Evaluating serum periostin and YKL-40 as biomarkers for airway remodeling and hyperresponsiveness in pediatric asthma^☆^

**DOI:** 10.1016/j.waojou.2024.100991

**Published:** 2024-10-30

**Authors:** Su Ji Kim, Youn Joo Choi, Man Yong Han, Il Tae Hwang, Hey-Sung Baek

**Affiliations:** a Department of Pediatrics, Kangdong Sacred Heart Hospital, Hallym University College of Medicine, Seoul, South Korea; b Department of Ophthalmology, Kangdong Sacred Heart Hospital, Hallym University College of Medicine, Seoul, South Korea; c Departments of Pediatrics, CHA Bundang Medical Center, CHA University School of Medicine, Seongnam, South Korea

**Keywords:** Periostin, Chitinase-like proteins (YKL-40), Asthma, Airway hyperresponsiveness

## Abstract

**Background:**

Periostin and human chitinase-3-like protein 1 (YKL-40) have been suggested to be involved in the development of airway fibrosis and remodeling. This study aimed to investigate the relationship between serum periostin levels and airway hyperresponsiveness (AHR) and between serum YKL-40 levels and AHR in children with asthma, comparing periostin as a marker for Th2 inflammation and atopy with YKL-40.

**Methods:**

The study involved children aged 6–15 years, comprising 75 with asthma and 29 healthy controls. We measured serum periostin and YKL-40 levels and performed exercise bronchial provocation tests, methacholine challenge tests, spirometry, and FeNO measurements.

**Results:**

Compared to the healthy controls, asthmatic children exhibited significantly elevated levels of periostin (86.7 [71.0–104.0] vs 68.3 [56.0–82.0] ng/mL; P = 0.006) and YKL-40 (29.0 [15.0–39.5] vs 27.7 [14.0–34.1] ng/mL; P = 0.034). The subgroup analysis revealed that periostin levels were significantly higher in the atopic asthma group than in the healthy controls (P = 0.003), but not in the non-atopic asthma group. YKL-40 levels were elevated in both the atopic and non-atopic asthma groups compared to healthy controls (P = 0.012 and P = 0.001, respectively). Serum periostin levels were significantly correlated with the postexerceise maximum percentage decrease in forced expiratory volume (FEV_1_), as well as with fractional exhaled nitric oxide (FeNO) and blood eosinophil counts, but showed no significant correlation with overall lung function. Conversely, serum YKL-40 levels were significantly linked to the Z score of FEV_1_ and AHR to methacholine but not with AHR to exercise or FeNO or blood eosinophil count.

**Conclusions:**

Periostin is linked to atopic asthma and correlates with exercise-induced bronchoconstriction, FeNO, and eosinophil counts, highlighting its role in Th2 inflammation. YKL-40 is a general asthma marker, indicating airway remodeling. These findings suggest that targeting these markers can improve personalized treatment strategies for pediatric asthma.

## Background

Asthma is a complex, heterogeneous airway disorder with multiple phenotypes, impacting its pathogenesis, history, and treatment.^1^^,^^2^ In asthma, airway remodeling encompasses the thickening of airway walls and submucosal tissue, along with smooth muscle hypertrophy and hyperplasia, leading to reduced lung function. Eosinophils play an unexpectedly significant role in airway remodeling and chronic inflammation.^3^ Airway hyperresponsiveness (AHR) is a key pathophysiological feature of asthma.^4^ Asthma airways are hyperresponsive, leading to increased sensitivity to inhaled methacholine, mannitol, and cold or dry air. The extent of AHR to these exposures is linked to asthma severity and airway inflammation.

Recognition of serum biomarkers, such as periostin, which has been identified as a biomarker for type 2 inflammatory airway disease, is valuable for diagnosing asthma and specifying its phenotypes or endotypes. Periostin has been recognized as a biomarker for type 2 inflammatory airway disease.^5^ YKL-40, a chitinase-3-like protein, is involved in inflammation and tissue remodeling. Previous research has shown that YKL-40 levels are elevated in individuals with asthma and are associated with asthma severity, subepithelial basement membrane thickening, and pulmonary function.^6^^,^^7^ Specifically, both periostin and YKL-40 have been suggested to be involved in the development of airway fibrosis and remodeling. Recent research in biologics and airway remodeling has garnered significant attention, with some studies showing full normalization of airflow obstruction, previously thought to be irreversible, in severe asthma patients treated with biologic therapies.^1^, ^2^, ^3^ Asthma is a very heterogeneous disease, and various types of airway remodeling may be associated with different phenotypes and endotypes. Our study, which focused on airway remodeling markers and their association with AHR in children with asthma, adds to the existing body of knowledge and emphasizes the need for further research to better understand the interplay between biomarkers and biologics treatments. Based on these findings, future research could potentially lead to more personalized and effective diagnostic and therapeutic approaches in the field of pediatric asthma. Our previous studies have shown that serum periostin levels correlate with AHR to methacholine,^8^ mannitol,^8^ and exercise^9^ among asthmatic children. Although various studies have examined the association between different biomarkers and AHR, few have simultaneously measured both periostin and YKL-40 to investigate their relationship with AHR in children with asthma. Therefore, this study aimed to investigate the relationship between serum periostin levels and AHR and between serum YKL-40 levels and AHR in children with asthma, comparing periostin as a marker for Th2 inflammation and atopy with YKL-40.

## Methods

### Subjects

Children were enrolled from February 2017 to November 2021. We organized patients with suspected asthma at their first visit. Asthma was diagnosed on the basis of the typical symptoms and confirmed variable expiratory airflow limitations which include positive bronchodilator test (increase in forced expiratory volume in 1 s [FEV_1_] of 12% or more after bronchodilator) or positive methacholine bronchial provocation tests (BPT) (less than 16.0 mg/mL of inhaled methacholine induced a 20% decrease in FEV_1_ [PC_20_])^10^ or positive exercise BPT (fall in FEV_1_ of >10% predicted).^11^ Asthma control status was assessed according to the Global Initiative for Asthma (GINA) guidelines. All asthmatic children had been well-controlled for at least 2–3 months before entering the study. We excluded children who had been treated with systemic corticosteroids for acute asthma exacerbation in the previous 6 months. In addition, patients with abnormal pulmonary disease detected on chest X-rays taken 1 month before the study were excluded.

The control group included children who came for routine health screenings or visited the outpatient clinic for vaccinations and matched by age and gender with the patient group. Only those without infection in the 2 weeks preceding the study were included. FeNO level of 20 parts per billion (ppb) or higher^12^ was used to exclude control group children at risk of atopy and subclinical eosinophilic inflammation. The skin prick test (SPT) was conducted for all participants, including both the asthma and control croups, against 11 inhalant allergens, including dog dander, cat dander, *Dermatophagoides pteronyssinus*, *D. farinae*, *Alternaria alternata*, birch, oak, ragweed, mugwort, and *Humulus japonicus* pollens (Allergopharma, Reinbek, Germany). Atopy was defined as positive findings in the SPT or the presence of at least 1 positive allergen-specific IgE test result (IgE ≥0.35 kU/L).

### Protocol

**Fig. 1:**
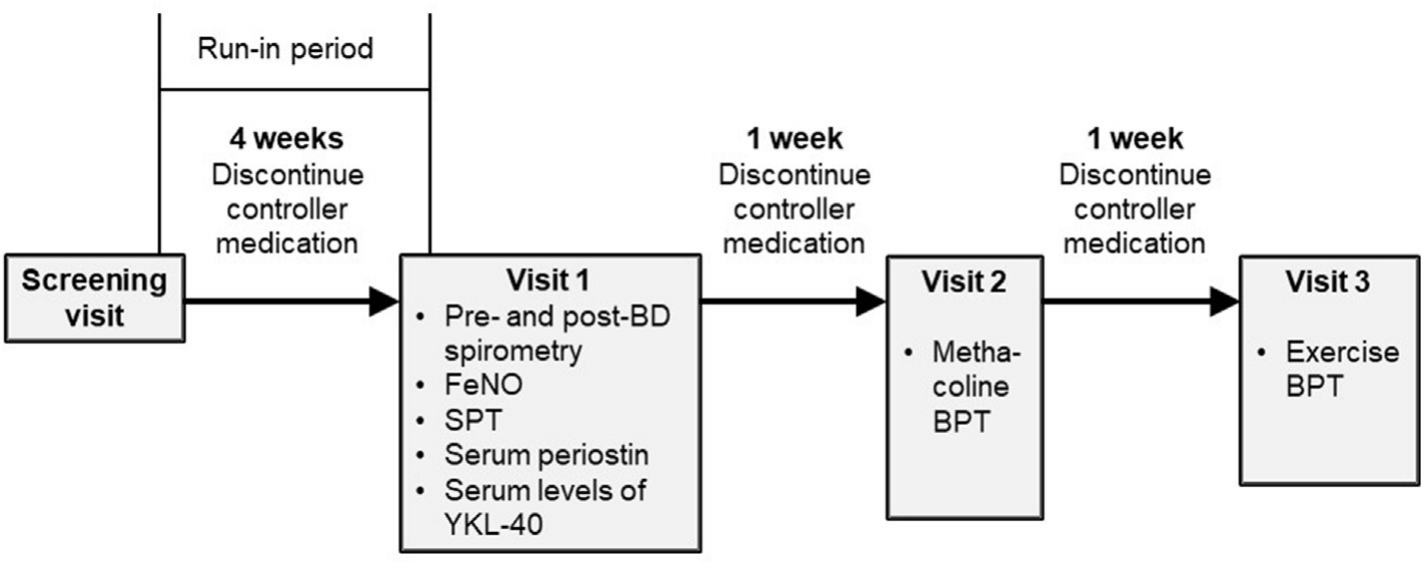
Schematic representation of the study design

The study design is illustrated in Fig. 1. Following a 4-week run-in period, patients with asthma visited our clinic 3 times during the study period. Patients were asked to discontinue inhaled corticosteroids (ICS) 1 month before testing/inclusion, to ensure that the baseline measurements of biomarkers were not influenced by the effects of ongoing ICS therapy. Patients who experienced asthma exacerbations and needed controller medications during the observation period were excluded from the study. Blood samples were taken during the initial visit, and physicians measured FeNO levels. Each participant underwent SPTs and pre- and postbronchodilator spirometry. A methacholine challenge test was conducted during the second visit. After an interval of at least 1 week, an exercise challenge BPT was performed during the third visit. The healthy control individuals also had 3 scheduled clinic visits. At the first visit, the physician measured FeNO levels, and each participant was examined with SPTs. The second visit included children without atopy or eosinophilic inflammation. Methacholine challenge BPTs were performed, and blood samples were collected. At the third visit, conducted at least 1 week later, exercise challenge BPTs were conducted.

Blood samples were stored at −70°C and serum levels of periostin, YKL-40, and total IgE were measured. Trained technicians performed spirometry and exercise challenge tests and measured FeNO levels. The study protocol and procedures were approved by the Medical Ethics Committee of Hallym University Kangdong Sacred Heart Hospital, Seoul, Korea. Written informed consent was obtained from all children and/or their parents. (IRB No. 2018-02-005).

### Methacholine BPT

AHR to methacholine was assessed using Probocholine (Masterlab®, Jaeger Co., Freiburg, Germany) in accordance with the American Thoracic Society (ATS) guidelines.^10^ Methacholine was delivered from a nebulizer using the dosimeter method. The provocation concentration that caused a 20% decrease in FEV_1_ from baseline was denoted as PC_20_. The provocation concentration that caused a 20% decrease in FEV1 from baseline is referred to as PC_20_.

### Exercise BPT

Exercise BPT was performed using a standardized protocol in accordance with the guidelines of the ATS^11^ on a motorized treadmill with a nose clip (LE 200 CE; Jaeger Co., Freiburg, Germany). The laboratory conditions were maintained at 22 °C with 40–50% humidity. The treadmill speed was incrementally increased to reach approximately 85% of the predicted maximum heart rate ([220–age] × 0.85), which was then sustained for 6 min. Spirometry tests were conducted 20 min and 5 min before the exercise challenge, and subsequently at 0, 3, 6, 10, 15, and 20 min post-exercise. The cut-off value for diagnosing exercise-induced bronchoconstriction —percent fall in FEV_1_ (Δ FEV_1_) ≥ 10% after exercise challenge test.^11^

### Fractional exhaled nitric oxide (FeNO)

FeNO levles were measured using a portable nitric oxide analyzer (NIOX VERO®; Aerocrine, Solna, Sweden) that operates at a 50 mL/s exhalation flow rate, with results expressed in parts per billion (ppb).^13^ This portable device's measurements align well with those of a stationary analyzer, as per the ATS guidelines^14^ Each participant underwent 2 measurements to reduce variability, maintaining an exhalation time of 10 s for each test.

### Measurement of serum biomarkers

Between 8:00 AM and 9:00 AM, blood samples were performed. Serum total and specific IgE levels against the same common allergens used for SPT were measured by immunoassay using the ImmunoCAP system (Phadia, Uppsala, Sweden). Serum levels of periostin were measured by Shino Test Corp. (Kanagawa, Japan) using enzyme-linked immunosorbent assay (ELISA) with 2 rat anti-human periostin mAbs (clone no. SS18A and SS17B). Serum YKL-40 levels were also measured by ELISA using the Human Chitinase 3-like 1 Quantikine ELISA Kit (catalog number DC3L10; R&D Systems, Inc., Minneapolis, MN, USA). Experiments were performed in accordance with to the manufacturer's instructions.

### Statistical analyses

All statistical analyses were performed using IBM SPSS Statistics 25.0 (IBM Corp.). Continuous data were expressed as means with standard deviations or medians with interquartile ranges (depending on data distribution). Dermographic data, including age and BMI, were compared using the Mann–Whitney U test, while sex was compared using the chi-square test. The Mann–Whitney U or Kruskal–Wallis tests were used to compare continuous variables between groups. χ^2^ tests was used to analyze categorical variables. Numerical parameters with non-normal distributions were log-transformed. Spearman's rho was calculated to assess the correlations between periostin and YKL-40 levels, lung function, and AHR.

## Results

### Characteristics of study subjects

In total, 108 participants were recruited for this study. The patient group consisted of 79 patients with asthma and the control group included 29 healthy individuals. Four asthmatic participants withdrew during the run-in period due to their inability to discontinue controller medications following asthma exacerbations. A total of 104 participants completed the study; the final analysis included 75 asthmatic patients and 29 healthy controls. All patients were classified as well-controlled according to^7^ guidelines for at least 2–3 months before inclusion in the study. Additionally, 56% of the asthma group were newly diagnosed and had not yet started ICS therapy.

The baseline characteristics of the study subjects, including the presence of allergic diseases, are shown in Table 1. Among 75 children with asthma, 54 (72%) had allergic rhinitis (AR) and 14 (18.7%) had atopic dermatitis (AD). No statistically significant differences were found in age, sex, or body mass index (BMI) between asthmatic and healthy children. The mean ages were 8.9 years in the asthma group and 8.6 years in the control group. The age range of the children in both the asthma and healthy control group was 6–15 years. Atopy was observed in 54 (72%) asthmatic children.

**Table 1:** Baseline characteristics of studied participants.

	Patients with asthma (n = 75)	Healthy controls (n = 29)	P value^a^
Age (y)	8.9 ± 2.3 (range 6–15)	8.6 ± 1.6 (range 6–15)	0.439
BMI (kg/m^2^)	18.9 ± 3.5	17.8 ± 1.9	0.162
Male/female sex			0.357^b^
Male, no. (%)	52 (69.3)	17 (58.6)	
Female, no. (%)	23 (30.7)	12 (41.4)	
Atopy (%)	54 (72%)	NA^c^	NA
Previous treatment			
No ICS (%)	42 (56)	NA	NA
With ICS (%)	33 (44)	NA	NA
Parental history of any allergic diseases, no. (%)	53 (70.7)	7 (24.1)	<0.001
Comorbid allergic diseases			
Allergic rhinitis symptoms in the previous 12 mo, no. (%)	54 (72)	NA	NA
Atopic dermatitis symptoms in the previous 12 mo, no. (%)	14 (18.7)	NA	NA

a Mann-Whitney U test.

b Chi-square test, the p-value compares the gender distribution between patients with asthma and healthy controls.

c Not Applicable. Control group marked with a symbol were selected to not have asthma or atopy, and assessment of comorbid allergic diseases was thus not applicable. Atopy was marked as NA since participants with FeNO levels >20 ppb, positive SPT results, or at least one positive allergen-specific IgE test result (IgE ≥0.35 kU/L) were excluded.

### Pulmonary function, FeNO, AHR to methacholine and exercise

In the asthma group, FEV_1_ and FEV_1_/forced vital capacity (FVC) ratios were lower than in the control group, while bronchodilator responses were significantly higher. (Table 2). The asthmatic group exhibited a significantly greater reduction in FEV_1_ following exercise compared to the healthy group. The mean PC_20_ in the asthma group was 1.94. The total IgE and peripheral blood (PB) eosinophil counts were significantly higher in patients with asthma than in healthy controls. FeNO levels were significantly higher in asthmatic patients compared to healthy controls.

**Table 2:** Differences in individual characteristics and pulmonary functions

	Patients with asthma (n = 75)	Healthy controls (n = 29)	P value
FEV_1_ (pred %)	87.6 ± 13.2	102.5 ± 9.9	<0.001
FVC (pred %)	95.4 ± 13.0	95.9 ± 9.7	0.051
FEV_1_/FVC ratio	0.825 ± 0.828	0.901 ± 0.121	<0.001
Postbronchodilator ΔFEV_1_ (pred %)	10.2 ± 9.2	2.4 ± 4.1	<0.001
Post-exercise maximum decrease in FEV_1_ (%)	17.9 ± 12.1	5.7 ± 4.1	<0.001
PC_20_ (mg/mL)	1.94 ± 2.48	NA	NA
Total IgE (IU/mL)	258.6 (64.7–699.8)	35.2 (12.6–62.4)	<0.001
PB eosinophil (/mL)	554.3 ± 254.4	143.8 ± 50.7	<0.001
FeNO (ppb)	28 (18–44.0)	10.0 (8.0–15.3)	<0.001

### Correlations between serum periostin levels and AHR, and between serum YKL-40 levels and AHR

**Fig. 2:**
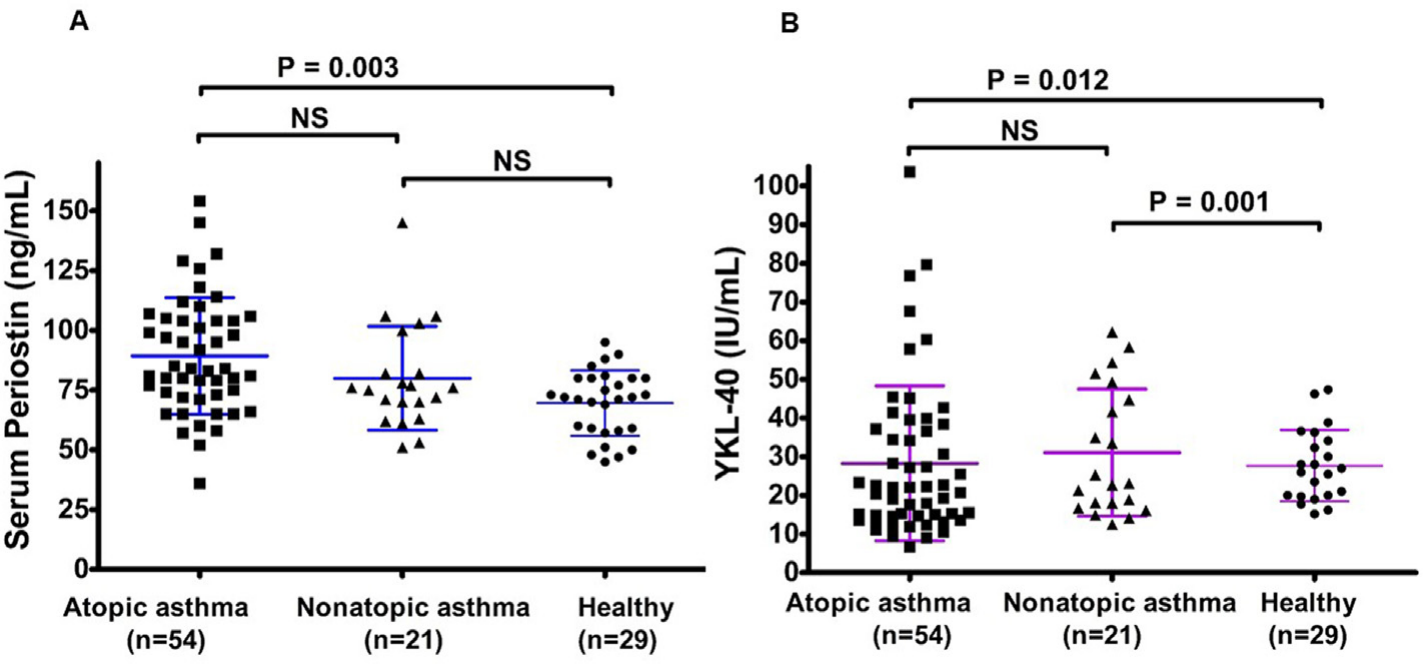
The serum periostin (A) and YKL-40 (B) levels in atopic asthma, non-atopic asthma, and healthy control groups

Compared to the healthy controls, asthmatic children exhibited significantly elevated levels of periostin (86.7 [71.0–104.0] vs 68.3 [56.0–82.0] ng/mL; P = 0.006) and YKL-40 (29.0 [15.0–39.5] vs 27.7 [14.0–34.1] ng/mL; P = 0.034). Analysis of serum periostin and YKL-40 levels across different subgroups revealed significant differences. Periostin levels were significantly higher in the atopic asthma group than in the healthy control group (P = 0.003). However, there was no significant difference between the non-atopic asthma and healthy control groups or between the atopic and non-atopic asthma groups. This suggests that periostin was more closely associated with atopy than with asthma. YKL-40 levels were significantly higher in the atopic asthma group compared to the healthy control group (P = 0.012) and also significantly higher in the non-atopic asthma group compared to the healthy controls (P = 0.001). However, no significant differences were observed between atopic and non-atopic asthma groups. This indicates that YKL-40 is elevated in those with asthma, irrespective of their atopic status. A detailed subgroup analysis is shown in Fig. 2.

Serum levels of periostin were significantly correlated with post-bronchodilator ΔFEV_1_, methacholine PC_20_, post-exercise maximum decrease in % FEV_1_, eosinophil counts in PB, and FeNO levels, but were not associated with lung function (Z FEV_1_ and FEV_1_/FVC ratio) (Table 3). Serum YKL-40 levels were significantly correlated with lung function (Z FEV1), post-bronchodilator Δ FEV_1_ (pred %), and methacholine PC20, but showed no significant association with the maximum post-exercise maximum decrease in % FEV_1_, FeNO, or PB eosinophil counts. Serum-specific IgE levels in *Dermatophagoides farinae* and *Dermatophagoides pteronyssinus* significantly correlated with periostin levels. However, serum total IgE and IgE levels against other allergens did not significantly correlate with periostin levels. Additionally, there was no significant correlation between the YKL-40 and serum-specific gE levels or total IgE levels (Table 3).

**Table 3:** Spearman's correlation coefficients among study participants

	Serum periostin level (ng/mL)		YKL-40 level (IU/mL)	
	R	P-value	r	P-value
Z FEV_1_	−0.068	0.571	−0.262	0.023
FEV_1_/FVC ratio	−0.038	0.754	−0.031	0.789
Postbronchodilator ΔFEV_1_ (pred %)	−0.284	0.015	−0.211	0.043
Methacholine PC20	−0.281	0.022	−0.294	0.017
Post-exercise maximum decrease in FEV_1_ (%)	0.358	0.022	0.215	0.102
PB eosinophils (/ml)	0.265	0.026	0.065	0.622
FeNO (ppb)	0.484	<0.001	0.118	0.336
Total IgE (IU/ml)	0.229	0.147	0.277	0.119

## Discussion

Our study showed that compared to healthy controls, periostin and YKL-40 levels significantly increased in asthmatic children. Periostin levels were significantly correlated with both AHR to methacholine and AHR to exercise, as well as with FeNO and PB eosinophils. In contrast, YKL-40 levels were significantly correlated only with AHR to methacholine but not with AHR to exercise FeNO, or PB eosinophils.

Periostin is a biomarker for identifying atopic asthma in children, and it is indicative of Th2-induced airway inflammation.^15^, ^16^, ^17^ In the present study, periostin levels in the serum were significantly higher in asthmatic patients than in healthy controls and were significantly correlated with both the PB eosinophil and FeNO levels, which was consistent with the data of previous studies.^15^^,^^16^ Jia et al^16^ identified periostin as a systemic biomarker for airway eosinophilia in asthmatic patients, suggesting its potential use in selecting patients for new asthma treatments targeting Th2 inflammation. Periostin was highly expressed in the lungs of patients with idiopathic pulmonary fibrosis,^17^ similar to that in patients with asthma. Two studies have shown that elevated levels of periostin in the serum and plasma of patients with idiopathic pulmonary fibrosis predicted decreased lung function after 6 months and 48 weeks, respectively.^18^^,^^19^ Although there are reports of associations between pulmonary function and periostin levels, data on the relationship between periostin and small airway function remain limited. However, in our study, periostin was not correlated with overall lung function, indicating that it reflects the specific inflammatory aspects of asthma. We found that serum periostin levels correlated with AHR to methacholine and AHR to exercise, but were not associated with lung function. Clinically, periostin is useful for phenotyping asthma, particularly for monitoring inflammatory responses and exercise-induced bronchoconstriction. This can help personalize treatment strategies based on individual inflammatory profiles and triggers. It should be used alongside other clinical assessments and biomarkers for comprehensive evaluation of pediatric asthma.

YKL-40 is involved in inflammation and tissue remodeling.^20^ Konradsen et al. demonstrated that YKL-40 levels were remarkably higher in children with severe therapy-resistant asthma than in healthy children and ichildren with controlled asthma following correction for genotype.^6^ The precise role of YKL-40 is still not fully understood; however, it has been repeatedly associated with airway obstruction in studies on asthma patients and with indicators of airway remodeling.^6^^,^^21^^,^^22^ In this study, serum YKL-40 levels were significantly correlated with lung function and AHR to methacholine but were not associated with AHR to exercise, FeNO levels, or PB eosinophil counts. The characteristics of bronchoconstriction induced by methacholine and exercise differ. Exercise triggers airway smooth muscle contraction through several intermediate mechanisms, including local and central neuronal reflexes and the release of inflammatory mediators like histamine, leukotrienes, and prostaglandins, leading to airway inflammation and constriction.^23^ In contrast, methacholine directly stimulates smooth muscle receptors in the airways, causing constriction without involving inflammation.^23^^,^^24^ Exercise tests correlate more strongly with the degree of airway inflammation and disease severity than methacholine challenge tests.^23^

Additionally, periostin levels were significantly higher in the atopic asthma group compared to the healthy control group, with no significant difference between non-atopic asthma and healthy controls, suggesting a closer association with atopy. This association may be attributed to the role of periostin in Th2 inflammation and IgE production. Woodruff et al^15^ demonstrated that periostin, encoded by the IL-13-responsive gene POSTN, could divide asthmatic Th2low phenotypes, with high-Th2 patients responding well to ICS. This supports our finding that periostin is more closely linked to atopic asthma, reflecting its specific association with Th2-driven inflammation.

In contrast, YKL-40 levels were elevated in both the atopic and non-atopic asthma groups as compared to healthy controls, indicating that it was generally more indicative of asthma, irrespective of the atopy status. YKL-40 is secreted by airway epithelial cells and macrophages and is involved in multiple inflammatory pathways beyond the Th2 axis. Tang et al.^25^ demonstrated that YKL-40 increases IL-8 production in human bronchial epithelial cells via MAPK (JNK and ERK) and NF pathways, leading to enhanced proliferation and migration of bronchial smooth muscle cells. This suggests that YKL-40 contributes to airway inflammation and remodeling by inducing IL-8 production, which play an active role in the pathophysiology of asthma. Létuvé et al.^26^ also found that YKL-40 stimulated IL-8 production in human macrophages, further supporting its role in the inflammatory response. These broad inflammatory actions may explain why YKL-40 serves as a general marker for asthma and may be linked to the fundamental mechanisms of airway remodeling and obstruction that are common to different asthma phenotypes. These findings highlight that, while periostin is strongly associated with atopy, YKL-40 is generally more indicative of asthma, which does not differentiate between atopic and non-atopic asthma.

This study had several limitations. Firstly, the sample size is limited. Secondly, our study cannot address the potential connection between our findings and poor asthma control, as we did not include patients who experience frequent asthma exacerbations. Third, the high prevalence of AR in asthmatic children suggests significant overlap between these conditions. Due to the absence of non-asthmatic AR control participants, it was challenging to delineate the specific impact of AR versus asthma on the elevated levels of periostin and YKL-40 as observed in our study. Additionally, we did not investigate the effect of ICS therapy on periostin levels, which would have been valuable for understanding its clinical utility when monitoring therapeutic responses. We also did not collect data on other respiratory medications, thus limiting our ability to assess their impact. Therefore, future studies should explore these aspects. Despite these limitations, our study provides valuable information on airway remodeling.

## Conclusions

We found that periostin was strongly associated with atopic asthma, correlating significantly with exercise-induced bronchoconstriction, FeNO levels, and blood eosinophil counts, thereby indicating its role in Th2-driven inflammation and hyper-responsiveness. In contrast, YKL-40 serves as a more general marker for asthma, indicating airway remodeling without differentiating between atopic and non-atopic asthma. Our research provides insights that may improve asthma management and treatment strategies by focusing on airway remodeling markers and their association with AHR in children with asthma. Understanding these interactions can lead to more personalized and effective treatment approaches for pediatric asthma management.

## Abbreviations

YKL-40: human chitinase-3-like protein 1; AHR: airway hyperresponsiveness, FeNO: factional exhaled nitric oxide; BPTs: bronchial provocation tests; FEV1: the maximum decrease in % forced expiratory volume; PB: peripheral blood.

## Availability of data and materials

The datasets used and/or analysed during the current study are available from the corresponding author on reasonable request.

## Authors' contributions

Su Ji Kim: conception and design of the study, collection of the data and analysis and interpretation of the data, and preparation of the manuscript.

Youn Joo Choi, MD: conception and design of the study, collection of the data and analysis and interpretation of the data, and preparation of the manuscript.

Il Tae, Hwang, MD, PhD: conception and design of the study, interpretation of the data, and preparation of the manuscript.

Man Yong Han, MD, PhD: conception and design of the study, interpretation of the data, and preparation of the manuscript.

Heysung Baek, MD, PhD: conception and design of the study, collection of the data and analysis and interpretation of the data, and preparation of the manuscript.

All authors read and approved the final manuscript.

## Ethics approval and consent to participate

All procedures were approved by the Medical Ethics Committee of Hallym University Kangdong Sacred Heart Hospital, Seoul, Korea, and all subjects and/or parents gave written informed consent. (IRB No. 2018-02-005).

## Authors’ consent for publication

All authors consent to the publication of this manuscript in the World Allergy Organization Journal.

## Funding

This research was supported by a grant no. 2019-14 from the Kangdong Sacred Heart Hospital Fund.

## Declaration of competing interest

The authors declare that they have no competing interests.
